# Isoprostanoid Plasma Levels Are Relevant to Cerebral Adrenoleukodystrophy Disease

**DOI:** 10.3390/life12020146

**Published:** 2022-01-20

**Authors:** Cinzia Signorini, Claudio De Felice, Thierry Durand, Jean-Marie Galano, Camille Oger, Silvia Leoncini, Joussef Hayek, Jetty Chung-Yung Lee, Troy C. Lund, Paul J. Orchard

**Affiliations:** 1 Department of Molecular and Developmental Medicine, University of Siena, 53100 Siena, Italy; 2 Neonatal Intensive Care Unit, Azienda Ospedaliera Universitaria Senese, 53100 Siena, Italy; c.defelice@ao-siena.toscana.it; 3 Institut des Biomolécules Max Mousseron (IBMM), UMR 5247, CNRS, Université de Montpellier, ENSCM, CEDEX 5, 34093 Montpellier, France; thierry.durand@umontpellier.fr (T.D.); jean-marie.galano@umontpellier.fr (J.-M.G.); camille.oger@umontpellier.fr (C.O.); 4 Child Neuropsychiatry Unit, Azienda Ospedaliera Universitaria Senese, 53100 Siena, Italy; silvia.leoncini@ao-siena.toscana.it (S.L.); associazionediateso@gmail.com (J.H.); 5 Pediatric Speciality Center “L’Isola di Bau”, Certaldo, 50052 Florence, Italy; 6 School of Biological Sciences, The University of Hong Kong, Hong Kong, China; jettylee@hku.hk; 7 Division of Pediatric Blood and Marrow Transplantation, University of Minnesota, Minneapolis, MN 55455, USA; lundx072@umn.edu (T.C.L.); orcha001@umn.edu (P.J.O.)

**Keywords:** cerebral adrenoleukodystrophy, fatty acids, hematopoietic stem cell transplantation, isoprostanes, N-acetyl-L-cysteine, neuroprostanes

## Abstract

Cerebral adrenoleukodystrophy (ALD) is a rare neuroinflammatory disorder characterized by progressive demyelination. Mutations within the *ABCD1* gene result in very long-chain fatty acid (VLCFA) accumulation within the peroxisome, particularly in the brain. While this VLCFA accumulation is known to be the driving cause of the disease, oxidative stress can be a contributing factor. For patients with early cerebral disease, allogeneic hematopoietic stem cell transplantation (HSCT) is the standard of care, and this can be supported by antioxidants. To evaluate the involvement of fatty acid oxidation in the disease, F_2_-isoprostanes (F_2_-IsoPs), F_2-_dihomo-isoprostanes (F_2_-dihomo-IsoPs) and F_4_-neuroprostanes (F_4_-NeuroPs)—which are oxygenated metabolites of arachidonic (ARA), adrenic (AdA) and docosahexaenoic (DHA) acids, respectively—in plasma samples from ALD subjects (*n* = 20)—with various phenotypes of the disease-were measured. Three ALD groups were classified according to patients with: (1) confirmed diagnosis of ALD but without cerebral disease; (2) cerebral disease in early period post-HSCT (<100 days post-HSCT) and on intravenous N-acetyl-L-cysteine (NAC) treatment; (3) cerebral disease in late period post-HSCT (beyond 100 days post-HSCT) and off NAC therapy. In our observation, when compared to healthy subjects (*n* = 29), in ALD (i), F_2_-IsoPs levels were significantly (*p* < 0.01) increased in all patients, with the single exception of the early ALD and on NAC subjects; (ii) significant elevated (*p* < 0.0001) amounts of F_2_-dihomo-IsoPs were detected, with the exception of patients with a lack of cerebral disease; (iii), a significant increase (*p* < 0.003) in F_4_-NeuroP plasma levels was detected in all ALD patients. Moreover, F_2_-IsoPs plasma levels were significantly higher (*p* = 0.038) in early ALD in comparison to late ALD stage, and F_4_-NeuroPs were significantly lower (*p* = 0.012) in ALD subjects with a lack of cerebral disease in comparison to the late disease stage. Remarkably, plasma amounts of all investigated isoprostanoids were shown to discriminate ALD patients vs. healthy subjects. Altogether, isoprostanoids are relevant to the phenotype of X-ALD and may be helpful in predicting the presence of cerebral disease and establishing the risk of progression.

## 1. Introduction

The pathogenic variant of the ATP-binding cassette subfamily D member 1 (*ABCD1*) gene is the cause of X-linked adrenoleukodystrophy (X-ALD) (OMIM ID: 300100) in most clinical cases. *ABCD1* encodes for a gene product that affects the transportation of very long-chain fatty acids (VLCFAs) to peroxisomes. The loss of *ABCD1* function implicates VLCFAs accumulation, and can lead to neurologic and adrenal dysfunction [1]. Cerebral X-ALD (cALD) is a severe form that generally affects young boys, is associated with cerebral inflammation and demyelination, and is generally progressive and lethal. The standard of care in arresting the progression of cerebral disease is by allogeneic hematopoietic stem cell transplantation (HSCT) [2,3].

Oxidative stress has been described to take part in the pathogenesis of ALD [4,5,6]. In addition, the link between oxidative stress and X-ALD [7,8,9] provided the rationale to use antioxidants for treating adrenomyeloneuropathy in murine model, as well as in a human pilot study [10]. N-acetyl-L-cysteine (NAC) is an antioxidant that can maintain glutathione levels [11] and is able to liberate protein-bound cysteine in plasma [12]. Moreover, NAC was reported to protect neurons from oxidative damage [13].

Recently, the mechanisms of NAC-induced antioxidant activity were investigated in X-ALD [14]. In this context, NAC may prove an important adjuvant treatment with allogenic HSCT in cerebral X-ALD [15,16].

In the brain, both harmful activities and toxic effects of fatty acids have recently been extensively examined [17], and an advanced mechanism to understand oxidative stress signaling, including the involvement of fatty acid oxidation (i.e., lipid peroxidation) were debated [18]. Undoubtedly, an indicator of oxidative stress is the measurement of prostaglandins from non-enzymatic oxidation of polyunsaturated fatty acid (PUFA), in particular, F_2_-isoprostanes (F_2_-IsoPs) from arachidonic acid (ARA). These have been evaluated in X-ALD [19]. However, in addition to F_2_-IsoPs, other isoprostanoids, such as F_2_-dihomo-isoprostanes (F_2_-dihomo-IsoPs) from adrenic acid (AdA) and F_4_-neuroprostanes (F_4_-NeuroPs) from docosahexaenoic acid (DHA), are formed simultaneously [20,21]. The presence of PUFA in the human brain is vital for normal neurological function [22] and the localization of different PUFAs can be different, i.e., AdA is primarily concentrated in the brain white matter and the grey matter is high in DHA, while ARA is found in both. In newborns, the most abundant PUFAs in the brain are DHA, ARA, and AdA. A marked increase is observed in DHA and AdA, which are also part of the VLCFA, in the period from 26 prenatal weeks to 8 postnatal years [23,24]. Thus, the generation of isoprostanoids of PUFAs appear to be relevant biomarkers in neurological diseases [25,26,27]. In particular, the plasma concentration of two F_4_-NeuroP isomers (i.e., 4-F_4t_-NeuroP and 10-F_4t_-NeuroP) [28,29,30] can be used to discriminate between different brain diseases and can be useful as neurological disease progression biomarkers [26,31,32], simultaneously with F_2_-dihomo-IsoPs and F_2_-IsoPs [27,31,33].

Interestingly, lipid peroxidation has been shown to be a constant and an early feature intimately linked to the pathophysiology of Alzheimer’s disease, as shown by F_2_-IsoP levels [34,35], and F_2_-IsoPs has been suggested as a possible predictor of the neurological disorder [34]. Furthermore, the roles of F_2_-IsoPs and F_4_-NeuroPs have been extensively analyzed as biomarkers of oxidative stress in neurodegenerative diseases [36]. Both IsoPs and NeuroPs have been used to monitor the occurrence of oxidative stress and in evaluating appropriate antioxidant therapies in brain pathologies [37]. The adequacy of isoprostanoids to evaluate brain damage has also been widely established in pediatric and neonatal conditions [38,39,40,41,42].

Currently, the relevancy of isoprostanoids in X-ALD in connection to fatty acid metabolism remains poorly understood. In the present study, we investigated the significance of F_2_-IsoPs, F_2_-dihomo-IsoPs, and F_4_-NeuroPs in X-ALD subjects, with or without cerebral disease, at different stages of treatment. In particular, we aimed to better understand the relevance of isoprostanoid formation in X-ALD and its association with cerebral disease.

## 2. Materials and Methods

### 2.1. Subjects

A total of twenty X-ALD patients (mean age 5.0 ± 2.2 years) took part in the study. All X-ALD patients were seen and evaluated at the University of Minnesota, Minneapolis, USA. The ALD patients were divided into three different groups based on disease and time post-HSCT: (1) patients with confirmed X-ALD diagnosis but without evidence of cerebral disease (*n* = 3); (2) patients with cALD in early period post-HSCT (<100 days) and receiving NAC treatment (*n* = 10); (3) patients with cALD in late period post-HSCT (beyond 100 days) and not on NAC therapy (*n* = 7).

In addition, a group of age-matched healthy control subjects (*n* = 29) were included in the study (admitted to the Child Neuropsychiatry Unit of the Azienda Ospedaliera Universitaria Senese, Siena, Italy, whose Head was J.H.). Written informed consent was obtained from all X-ALD subjects or their guardians on University of Minnesota Blood and Marrow Transplant (BMT) Program Protocols approved by the University Institutional Review Board, in accordance with the Declaration of Helsinki. Patients were treated on myeloablative transplant protocols, using NAC (70 mg/kg every 6 h) through 100 days after transplantation [3]. For all of the involved subjects, the study was carried out in accordance with the rules expressed in the Declaration of Helsinki Ethical Principles for Medical Research involving Human Subjects (Brazil, 2013).

### 2.2. Plasma Sample Preparation

Platelet poor plasma samples were obtained by centrifugation (2400× *g* for 15 min at 4 °C) of blood aliquots collected in heparinized tubes. As an antioxidant preservative, butylated hydroxytoluene (Merck KGaA, Darmstadt, Germany) (90 μM prepared in ethanol) was added to each plasma samples and stored at −70 °C until analysis.

### 2.3. Sample Preparation and Analysis

Fifty microliters of internal standard tetra-deuterated prostaglandin F_2α_ (PGF_2α_-d_4_) (100 pg/10 μL prepared in ethanol) was added to each plasma sample (0.5–1.0 mL). After mixing, each sample was purified with two kinds of solid-phase extraction (SPE) cartridges, C_18_ (500 mg Sorbent per Cartridge, 55–105 µm Particle Size, 6cc, Waters, Milford, MA, USA), and NH_2_ (500 mg Sorbent per Cartridge, 55–105 μm Particle Size, 6cc, Waters, Milford, MA, USA) cartridges, respectively.

The C_18_ cartridge was preconditioned with 5 mL methanol and 5 mL water, and then loaded with the sample. Thereafter, it was washed with 10 mL acidic water (pH 3) and 10 mL water: acetonitrile (85:15, *v*/*v*) and eluted with 5 mL of hexane: ethyl acetate: propan-2-ol (30:65:5 *v*/*v*/*v*). Subsequently, it was loaded on NH_2_ cartridge that was preconditioned with 5 mL hexane, and cleaned in the following order: 10 mL of hexane: ethyl acetate (30:70, *v*/*v*), 10 mL acetonitrile: water (9:1, *v*/*v*) and 10 mL acetonitrile, with the final elute consisting of 5 mL ethyl acetate: methanol: acetic acid (10:85:5, *v*/*v*/*v*).

The collected NH_2_ eluate was evaporated under nitrogen gas at 40 °C and derivatized. In the derivatization process, the carboxylic group was converted into pentafluorobenzyl ester by incubating 40 μL pentafluorobenzyl bromide (10% in acetonitrile) and 20 μL diisopropylethylamine (10% in acetonitrile) at 40 °C for 45 min. At the end of the reaction, the samples were evaporated under nitrogen gas, and the hydroxyl groups were converted to trimethylsilyl ethers by incubating 50 μL of N,O-bis (trimethylsilyl) trifluoroacetamide and 5 μL of diisopropylethylamine (10% in acetonitrile) for 1 h at 45 °C [26].

The derivatized sample (2 μL) was analyzed by a gas chromatography/negative ion chemical ionization tandem mass spectrometry (GC/NICI–MS/MS). The gas chromatograph (Trace GC Thermo/Finnigan, San Jose, CA, USA) was set at splitless injection mode for 2 min and the oven temperature was 175 °C, increasing to 270 °C (30 °C/min). Helium was used as the carrier gas (1 mL/min) and the chromatographic separation was performed using a SPB 1701 GC capillary column (30 m × 0.25 mm i.d., 0.25 μm film thickness, Supelco, Bellafonte, PA, USA). The reagent gas for the chemical ionization was methane set to 2.0 mL/min flow rate. The tandem mass spectrometry (MS/MS, Polaris Q Thermo/Finnigan, San Jose, CA, USA) was set in negative ion mode for quantification of precursor to products ions at: *m*/*z* 569→299 for F_2_-IsoPs (15-F_2t_-IsoP isomer) [43,44], *m*/*z* 593→323 for F_4_-NeuroPs, [26,43], *m*/*z* 597→327 for F_2_-dihomo-IsoPs [42], and *m*/*z* 573→303 for the internal standard PGF_2α_-d_4_ [26,42]. The quantitation of each isoprostanoid was determined by relating the peak area of each isoprostanoid to the deuterated internal standard peak area of the calibration curves constructed.

### 2.4. Chemical Synthesis of 4-F_4t_-NeuroP, 10-F_4t_-NeuroP, and F_2t_-dihomo-IsoPs Reference Molecules

F_4_-NeuroPs and F_2_-dihomo-IsoPs reference molecules were synthesized in-house by Institut des Biomolécules Max Mousseron (Montpellier, France) [42,45,46,47].

**Scheme 1:**
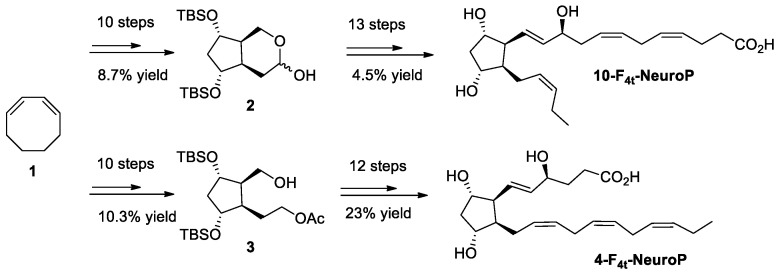
Schematic representation of the steps of chemical synthesis for of 4- and 10-F4t-NeuroPs [32].

The synthesis of the two series of 4- and 10-F_4t_-NeuroPs were described by our group in previous work [45,46,47]. The two compounds were obtained through20–22 steps of synthesis from commercially available 1,3-cyclooctadiene **1**. As an example of the synthetic work, Scheme 1 describes the synthesis of the 4- and the 10-F_4t_-NeuroP.

In brief, the two key bicyclic intermediates, **2** and **3,** were obtained in 10 steps, yielding 8.7% and 10.3%, respectively. The introduction of α and ω chains were performed by using regioselective protections/deprotections, oxidations, Wittig elongation, and cross metathesis coupling reactions as the main steps for the 10-F_4t_-NeuroP [47]. The final step was the saponification of the methyl esters in the presence of LiOH to obtain free acids. The 4-F_4t_-NeuroP was obtained starting from intermediate **3** in 12 more steps of synthesis, giving 23% yield after optimizations, while 10-F_4t_-NeuroP and its C10-epimer were obtained in 13 steps from the intermediate 2 [46].

**Scheme 2:**
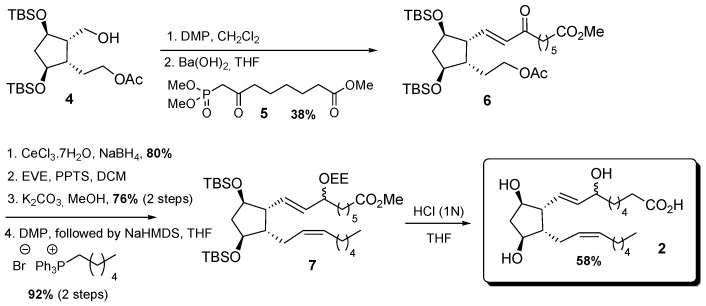
Schematic representation of the steps of synthesis of *ent*-7(*RS*)-F_2t_-dihomo-IsoP **2**. Symbols, 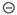 and 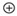, indicates the charge of the atoms (Br^-^ and P^+^, from phophonium salt) [42].

Starting from the mono-acetate **4**, the introduction of the α chain was performed by a Horner-Wadsworth-Emmons (HWE) reaction with the methyl 8-(dimethyloxyphosphoryl)-7-oxooctanoate **5**, obtained by condensation between dimethyl methyl phosphonate anion and dimethyl pimelate. The reduction in the α, β unsaturated ketone **6**, protection of the allylic hydroxyl group with ethoxyethyl vinyl ether, followed by successive deprotection of the acetate function in basic conditions, oxidation in mild conditions, and final Wittig reaction using commercial hexylphosphonium bromide, led to the formation of the ω chain of **7**. The ent-7(*RS*)-F_2t_-dihomo-IsoP 2 was obtained after a final one-pot-silylated ether deprotection and methyl ester hydrolysis in the presence of **1** N HCl (Scheme 2).

**Scheme 3:**
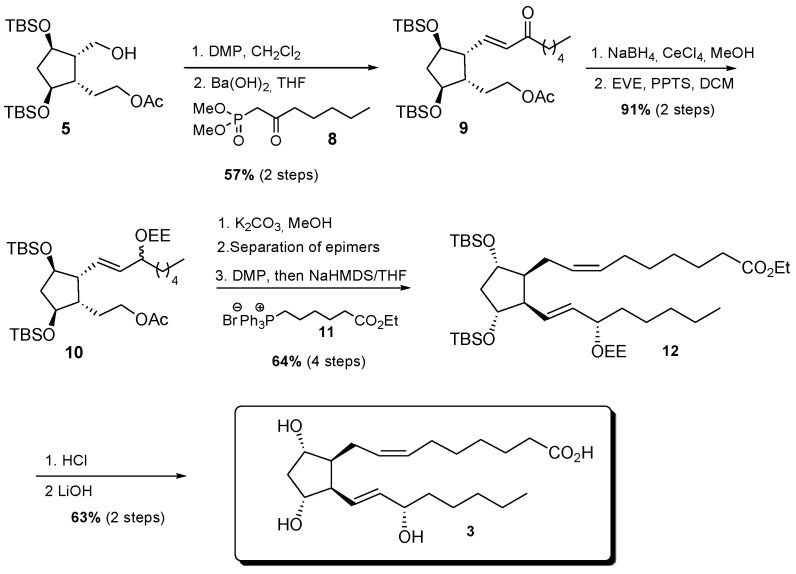
Schematic representation of the steps of synthesis of 17-F_2t_-dihomo-IsoP **3.** 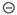 and 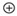, indicates the charge of the atoms (Br^-^ and P^+^, from phophonium salt) [42].

For 17-F_2t_-dihomo-IsoP synthesis, it was obtained starting from mono-acetate **4** in nine steps, using commercially available dimethyl-2-oxo-heptylphosphonate 8 and (7-ethoxy-7-oxoheptyl)triphenyl phosphonium bromide **11**, prepared in one step, and 93% yield from ethyl 7-bromo heptanoate. The enone **9** was obtained with 57% yield after Dess-Martin oxidation of alcohol **5** and HWE olefination with the β-keto phosphonate **8** and barium hydroxide. Enone **9** reduction was realized under Luche conditions, and the corresponding 1:1 epimer mixture was subsequently protected as ethoxy ether **10**, with 91% yield over two steps. The lower chain was introduced after saponification of the acetate group, followed by oxidation, separation of the two epimers at C17, then a Wittig elongation, using the previously synthesized phosphonium salt **11**, NaHMDS in THF, in 64% yield after four steps. Final acid cleavage of silylated ether **12**, followed by ethyl ester saponification, allowed the access to 17-F_2t_-dihomo-IsoP **3** with 63% yield after two steps (Scheme 3).

### 2.5. Data Analysis

All variables were tested for normal distribution (D’Agostino–Pearson test). The differences between groups were evaluated by Mann–Whitney test (independent samples), and multiple comparisons were carried out by Bonferroni multiple comparison test, or by one-way analysis of variance (ANOVA). The efficiency of isoprostanoids (F_2_-IsoPs, F_4_-NeuroPs, and F_2_-dihomo-IsoPs) in discriminating ALD from healthy control subjects was evaluated using receiver operating characteristic (ROC) curve analysis. A two-tailed *p* < 0.05 was considered to indicate statistical significance. The MedCalc ver. 12.0 statistical software package (MedCalc. Software, Mariakerke, Belgium) was used for the data analysis.

## 3. Results

The GC/NICI–MS/MS detection of 15-F_2t_-IsoPs, 4-F_4t_-NeuroP, 10-F_4t_-NeuroP, *ent*-7(RS)-F_2t_-dihomo-IsoP, and 17-F_2t_-dihomo-IsoP was carried out in all plasma samples that include the three distinct groups of X-ALD subjects and healthy subjects. The quantification of F_2_-IsoPs, F_4_-NeuroPs, and F_2_-dihomo-IsoPs in circulation are useful to consider in representing oxidative damage in these areas of the brain.

**Figure 1:**
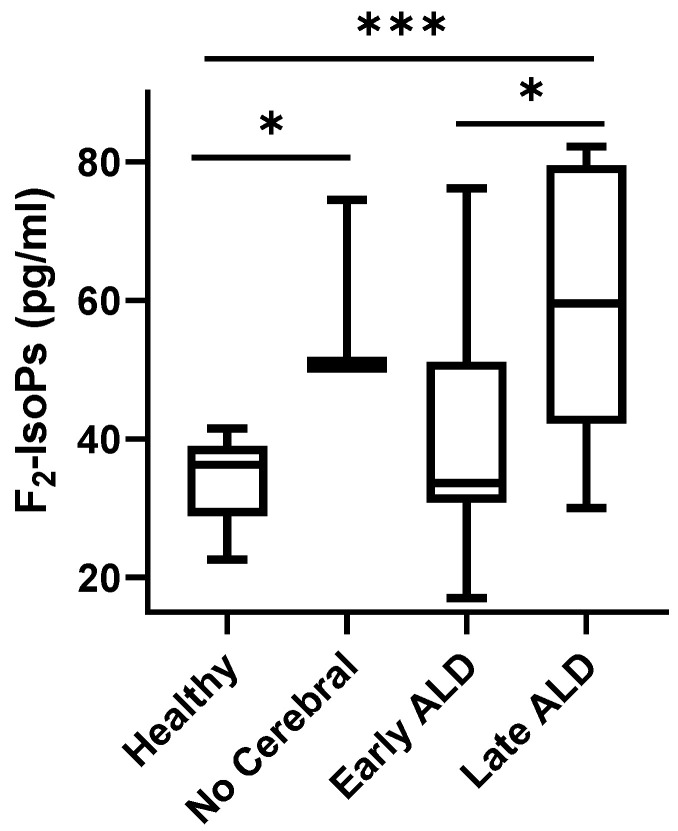
F_2_-IsoP plasma levels in ALD patients. Data are expressed as medians (columns), bars are minimum and maximum, and boxes represent inter-quartile ranges (25th–75th percentiles). Asterisks indicate significant pairwise tests (multiple comparison ordinary one-way ANOVA); * (0.01 ≤ *p* ≤ 0.03); *** *p* = 0.0001. Legend: healthy, healthy control group; no cerebral, confirmed ALD diagnosis but lack of cerebral disease; early ALD, ALD with cerebral disease in early period post-HSCT and on N-acetyl-L-cysteine (NAC) treatment; late ALD, ALD with cerebral disease in late period post-HSCT and off NAC therapy.

In all ALD patients, F_2_-IsoPs plasma levels (median, 46.85; 95% CI for the median 33.02 to 60.10) were significantly higher than in healthy control subjects (median, 36.3; 95% CI for the median 30.70 to 38.02) (*p* = 0.0041). In particular, significantly elevated amounts of plasma F_2_-IsoPs were found in two ALD sub-groups: (i) a diagnosis of X-ALD but lacking cerebral disease and (ii) cALD in late period post-HSCT, when patients were not receiving NAC. F_2_-IsoPs plasma levels were similar between the healthy control group and the cerebral X-ALD group receiving NAC early post-HSCT (Figure 1).

**Figure 2:**
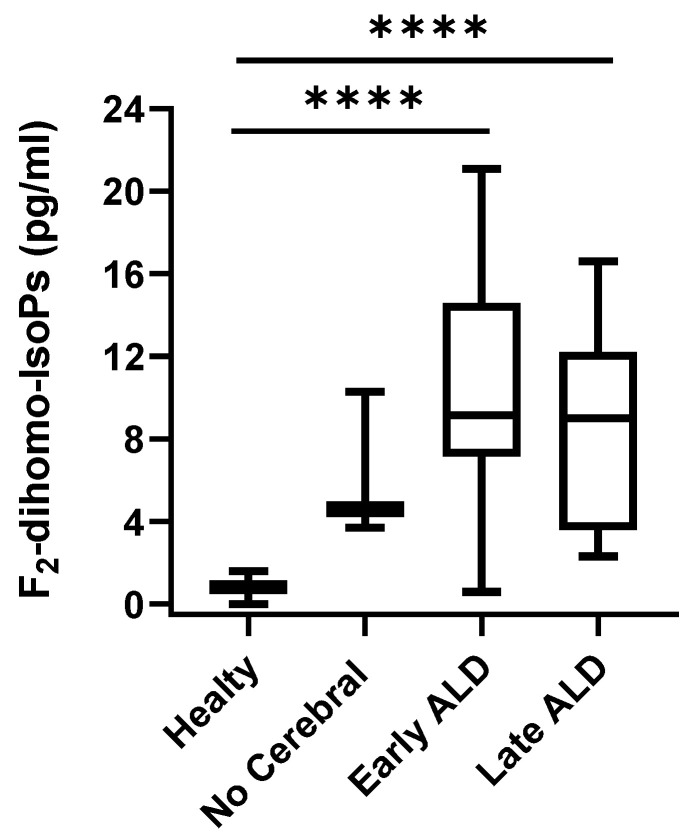
F_2_-dihomo-IsoP plasma levels in X-ALD patients. F_2_-dihomo-IsoP amounts were reported as the sum of the contents of ent-7(*RS*)-F_2t_-dihomo-IsoP and 17-F_2t_-dihomo-IsoP. Data are expressed as medians (columns), bars are minimum and maximum, and boxes represent inter-quartile ranges (25th–75th percentiles). Asterisks indicate significant pairwise tests (multiple comparison ordinary one-way ANOVA); **** *p <* 0.0001. Legend: healthy, healthy control group; no cerebral, confirmed ALD diagnosis but lack of cerebral disease; early ALD, ALD with cerebral disease in early period post-HSCT and on N-acetyl-L-cysteine (NAC) treatment; late ALD, ALD with cerebral disease in late period post-HSCT and off NAC therapy.

A significant difference (*p* < 0.0001) was found between F_2_-dihomo-IsoPs, representing AdA oxidation, plasma levels of ALD patients (median, 8.85; 95% CI for the median 6.23 to 11.76) and healthy subjects (median, 0.80; 95% CI for the median 0.60 to 1.02). With reference to ALD sub-groups, plasma levels of total F_2_-dihomo-IsoPs were significantly increased in both the early and late post-HSCT patients (currently receiving or not currently receiving NAC, respectively) compared to the healthy control subjects. However, no significant differences were shown for F_2_-dihomo-IsoPs when healthy control subjects were compared to the X-ALD population without cerebral disease (Figure 2).

**Figure 3:**
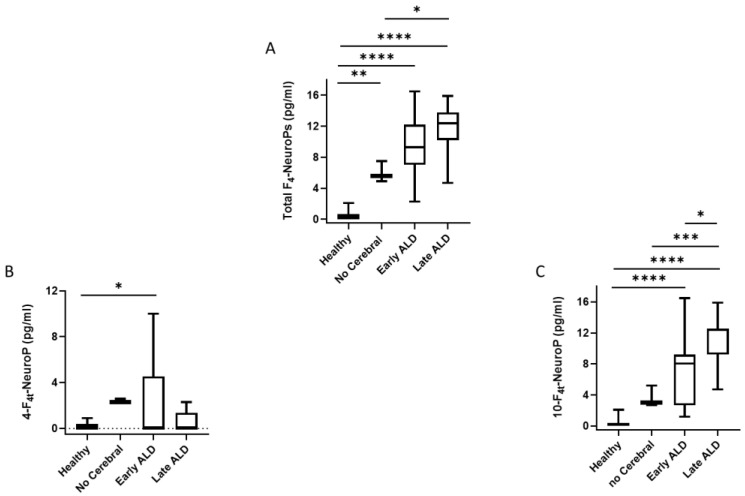
Total F_4_-NeuroPs, 4-F_4t_-NeuroP and 10-F_4t_-NeuroP plasma levels in ALD patients. Data are expressed as medians (columns), bars are minimum and maximum, and boxes represent inter-quartile ranges (25th–75th percentiles). Asterisks indicate significant pairwise tests (multiple comparison ordinary one-way ANOVA). Panel (**A**), * *p* = 0.012; ** *p* = 0.003; **** *p*< 0.0001. Panel (**B**), * *p* = 0.014: Panel (**C**), * *p* = 0.014; *** *p* = 0.0004; **** *p* < 0.0001. Legend: healthy, healthy control group; no Cerebral, confirmed ALD diagnosis but lack of cerebral disease; early ALD, ALD with cerebral disease in early period post-HSCT and on N-acetyl-L-cysteine (NAC) treatment; late ALD, ALD with cerebral disease in late period post-HSCT and off NAC therapy.

For total F_4_-NeuroPs (sum of 10-F_4t_-NeuroP and 4-F_4t_-NeuroP), the detected concentration in ALD patients (median, 9.85; 95% CI for the median 7.65 to 12.25) was higher than in healthy controls (median, 0.40; 95% CI for the median 0.00 to 0.50) (*p* < 0.0001). When total F_4_-NeuroPs were analyzed in ALD sub-groups, F_4_-NeuroPs levels were significantly higher in patients that did not have cerebral disease, as well as those post-HSCT on NAC therapy (early ALD), and post-HSCT off NAC therapy (late ALD), in comparison to the control group. A significant difference between post-HSCT off NAC therapy (late ALD) and lack of cerebral disease groups was also found (Figure 3A). Plasma levels of both the F_4_-NeuroP isomers analyzed (i.e., 10-F_4t_-NeuroP and 4-F_4t_-NeuroP) were significantly higher in post-HSCT on NAC therapy group in comparison to the healthy population (Figure 3B,C). Otherwise, only 10-F_4t_-NeuroP was shown to be increased in the remaining ALD (early and late) groups (Figure 3C). The detected amounts of the single F_4_-NeuroP isomers (i.e., 10-F_4t_-NeuroP or 4-F_4t_-NeuroP) did not statistically differ between the ALD patients with a lack of brain damage and the healthy subjects (Figure 3B,C).

Receiver operating characteristic (ROC) curves indicate that plasma levels of F_2_-IsoPs, F_2_-dihomo-IsoPs, total F_4_-NeuroPs, 10-F_4t_-NeuroP, and 4-F_4t_-NeuroP could discriminate between the ALD population (lack of cerebral disease, or with cerebral disease in early period post-HSCT on NAC therapy, or cerebral disease further from HSCT off NAC) and the healthy control subjects. All of the isoprostanoids were shown to be able to significantly discriminant ALD versus healthy control subjects, on the basis of the area under the ROC curve (AUC) value, including F_2_-dihomo-IsoPs (cut-off value > 0.2 pg/mL; sensitivity 100%; specificity 13.79%), total F_4_-NeuroPs (cut-off value > 2.2 pg/mL; sensitivity 100%; specificity 100%), and 10-F_4t_-NeuroP (cut-off value > 0.85 pg/mL; sensitivity 100%; specificity 96.55%) (Table 1).

## 4. Discussion

Accumulation of VLCFAs is a distinct feature of X-ALD, and oxidative damage has been strongly suggested to be an early driving cause of the X-ALD pathogenesis [5,6,8,48,49]. Although hexacosanoic acid (C26:0) is the main VLCFA in the accumulation of cerebral X-ALD, PUFAs should also be considered in the disease progression. PUFAs are prone to oxidation [25,50], and the resulting mix of metabolites, including the isoprostanoids, are considered biomarkers of certain neurological diseases, but hypothetically, they may be bioactive in the brain. Furthermore, it is shown that 4-F_4t_-NeuroP possess antiarrhythmic, anti-VIDD (ventilator-induced diaphragm dysfunction), neuroprotective, and anti-inflammatory properties [51,52]. Among the oxidized PUFA metabolites, aldehydes are thought to be potentially toxic, whereas data from isoprostanoid studies are less clear in terms of modifying the disease pathogenesis.

Accordingly, it is reasonable to use antioxidant treatment in clinical practice for cerebral X-ALD [10,14,53,54] to control PUFA oxidation, which is considered a common feature in neurological disease [25,55], including demyelinating diseases [56], as well as in brain atrophy [57]. In this study, it is apparent that the levels of isoprostanoids are increased in X-ALD and are affected by HSCT and antioxidant treatment. Thus, it can be deduced that antioxidant agents may be helpful, even if no cerebral disease is present.

In this preliminary trial, despite a small number of subjects without evidence of cerebral disease, we aimed to investigate the levels of isoprostanoids (i.e., F_2_-IsoPs, F_2_-dihomo-IsoPs, and F_4_-NeuroPs) that can reflect distinctive etiology within the brain matter [24,58]. As aforementioned, ARA and DHA are well known to be essential constituents of the central nervous system [23], and ARA has a function as the immediate precursor for AdA that is particularly enriched in myelin lipids [23,59]. PUFAs, essential components of the eukaryotic cell membrane, participate in the regulation of cell signaling pathways and act both as precursors of inflammatory and pro-resolving mediators [60]; the role of resolvins (a group of PUFA-derived proresolving mediators) in neurodegenerative diseases has been reviewed [61]. Apart from this, as noted, PUFAs serve as precursors of isoprostanes and neuroprostanes. ARA is esterified in membranes and it is a ubiquitous component of mammalian cell membranes. The increase in F_2_-IsoP amounts represent the occurrence of cellular oxidant injury. AdA- and DHA-derived isoprostanoids (F_2_-dihomo-IsoPs and F_4_-NeuroPs, respectively) have shown to be potential biomarkers for brain oxidative damage in different neurological diseases [26,31,41,42,43,44]. Usually, the evaluation of free isoprostanoids without considering the precursor content, i.e., PUFA, in biological fluids is considered a robust method to view in vivo oxidative stress [62]. However, recent evidence showed, aside from being biomarkers of oxidative stress, that they are also considered lipid mediators that are capable of participating in intracellular signaling [21]. Thus, isoprostanoid detection could be useful as a means to investigate the involvement of white and gray brain matter reflecting the pathogenic mechanisms of the development of cerebral X-ALD, and potentially as a biomarker related to the response to therapeutic interventions.

F_2_-IsoPs have been recognized as a biomarker of in vivo oxidative stress. Our study showed F_2_-IsoP plasma levels were not elevated in cALD patients when receiving NAC treatment, in comparison to control subjects. Such a result suggests that NAC, in the dose and schedule, was sufficient to counteract the oxidative stress. As an additional relevant point, among all isoprostanoids, F_2_-IsoPs appear to be the most sensitive for the antioxidant effect of NAC treatment. This result is in line with the assumption that F_2_-IsoPs represent an indicator of the systemic oxidative stress condition involving different tissue and biological fluids [63,64,65]. These observations suggest that the use of additional antioxidants are important in the early stages of the transplant process, at least regarding the hindering of oxidative stress that can represent pro-inflammatory factors.

F_2_-dihomo-IsoP and F_4_-NeuroP levels may be more reflective to neurological damage resulting from oxidative stress [25,66]. AdA, the PUFA precursor for F_2_-dihomo-IsoPs, has been shown to be present in several organs and tissues [67], and is highly concentrated in the myelin within the white matter. It has been proposed that products of AdA oxidation may serve as potential markers of free radical damage to the myelin in the human brain [57,68]. Thus, F_2_-dihomo-IsoPs are thought to be very low in the white matter at homeostasis within a healthy brain, while they are increased in neurological diseases related to oxidative damage. Our findings, therefore, seem consistent with the neuropathology of cerebral X-ALD, as it is characterized by the progressive degeneration of the cerebral white matter [69]. Importantly, within the myelin sheath of the human brain, AdA is more abundant than DHA or ARA [58,59]. An analysis of F_2_-dihomo-IsoPs and F_4_-NeuroPs, as biomarkers of lipid peroxidation of the nervous system, was performed as a means to evaluate clinical interventions in pathologies associated with the nervous system [66]. In our study, like F_2_-dihomo-IsoPs, 4-F_4t_-NeuroP and 10-F_4t_-NeuroP were not elevated in patients with X-ALD without cerebral disease, but when considered as total F_4_-NeuroPs (4-F_4t_-NeuroP + 10-F_4t_-NeuroP), they were augmented. Thus, the formation of the whole family of F_4_-NeuroP isomers, deriving from the oxidation of DHA, may provide an ability to define the phenotype of the disease. Interestingly, F_4_-NeuroPs, in particular 10-F_4t_-NeuroP, appear to be the isoprostanoid able to show a significant difference between the late period after HSCT and the ALD stage without cerebral disease. Moreover, isoprostanoids may define the effectiveness of anti-oxidant therapy. In addition, it should be noted that theoretically, 10-F_4t_-NeuroP cannot further oxidized [21], whereas 4-F_4t_-NeuroP can form hydroxyhexanal if the oxidation rate is not controlled [70], further suggesting the importance of 10-F_4t_-NeuroP as an oxidative damage biomarker in ALD. Thus, the detection of a panel of different isoprostanoids appear to show promise in providing important information of cerebral oxidative status.

Overall, the relevance of isoprostanoid plasma levels in evaluating brain tissue degeneration has been observed in different neurological conditions [25,26,57,71,72], and a model of evaluating white and grey brain matter lipid peroxidation by plasma biomarkers has been recently reported in early Alzheimer’s disease [57]. Moreover, low levels of plasma F_4_-NeuroPs, including 4-F_4t_-NeuroP, 10-F_4t_-NeuroP, and high levels of F_2_-dihomo-IsoPs, were shown to be associated with the risk of age-related macular degeneration [73].

Furthermore, the relevance of F_2_-dihomo-IsoPs, F_4_-NeuroPs, and F_2_-IsoPs to the disease severity was also shown in a murine model of infantile globoid cell leukodystrophy (GLD, also known as Krabbe disease) [27], another genetic demyelinating disease [74]. Thus, evidence is accumulating to hypothesize a common pathway, involving isoprostanoids, in assessing demyelinating disorders.

## 5. Conclusions

Our results strongly suggest that ARA, AdA, and DHA undergo in vivo non-enzymatic oxidation in X-ALD and show the high specificity of F_2_-dihomo-IsoPs and F_4_-NeuroPs in the evaluation of the oxidative state of PUFAs in case of pathology involving the brain.

**Figure 4:**
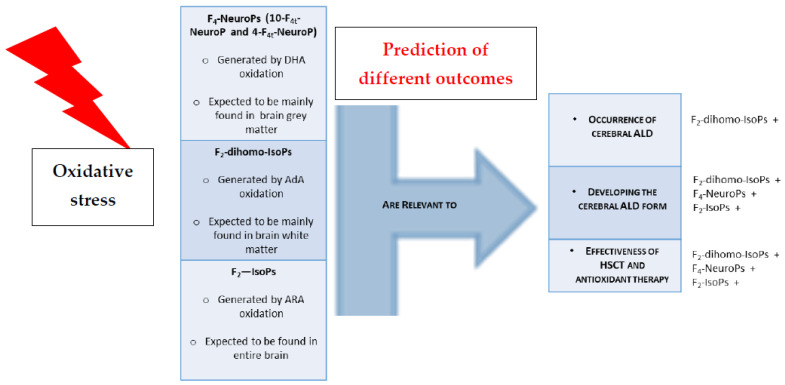
Isoprostanoids relevance to ALD clinical feature. Oxidative damage of PUFAs leads to formation of isoprostanoids that are predictors of ALD clinical outcome. Legend: +indicate isoprostanoid efficacy in discriminating the clinical condition.

Although oxidative stress may not prove to be the initiating factor of cerebral ALD, the mechanisms of oxidative stress may certainly contribute to the toxic effects of products that accumulate from the altered metabolism of VLCFA. Overall, our data suggest that isoprostanoids may prove important X-ALD biomarkers, and could potentially be helpful in predicting the development of cerebral disease and/or therapeutic efficacy. They might be useful to define (a) who did/did not have ALD, (b) who is developing the cerebral form, and (c) the effectiveness of the therapy (Figure 4).

## Figures and Tables

**Table 1:** Discriminating power for isoprostanoids (F_2_-IsoPs, F_2_-dihomo-IsoPs, total F_4_-NeuroPs, 10-F_4t_-NeuroP, and 4-F_4t_-NeuroP) content: summary of receiver operating characteristic (ROC) curve analysis.

**Entire ALD Group** ***vs*** **Healthy Control**	**Analysis**	**Area under the ROC Curve**	**Standard Error**	**95% C.I.**	***p* Value**
	F_2_-IsoPs	0.743	0.0830	0.598 to 0.857	0.0041
	F_2_-dihomo-IsoPs	0.9664	0.0330	0.871 to 0.997	<0.0001
	Total F_4_-NeuroPs	1.000	0.000	0.927 to 1.000	<0.0001
	10-F_4t_-NeuroP	0.997	0.00419	0.921 to 1.000	<0.0001
	4-F_4t_-NeuroP	0.602	0.0866	0.452 to 0.739	0.023

Data related to the area under the curve, relative standard error, 95% confidence interval, and significance (*p* value) are reported for each investigated variable (F_2_-IsoPs, F_2_-dihomo-IsoPs, total F_4_-NeuroPs, 10-F_4t_-NeuroP, and 4-F_4t_-NeuroP). Legend: C.I., interval of confidence, ROC, receiver operating characteristic curve.

## Data Availability

Data are available in the individual medical record of each patient.
